# Deciphering the axonal transport kinetics of neurofilaments using the fluorescence photo-activation pulse-escape method

**DOI:** 10.1186/1471-2202-15-S1-P132

**Published:** 2014-07-21

**Authors:** Yinyun Li, Anthony Brown, Peter Jung

**Affiliations:** 1 Department of Physics and Astronomy, Ohio University, Athens, OH 45701, USA; 2 III Institute of Physics-Biophysics, Georg-August-University Goettingen, Goettingen, 37077, Germany; 3 Department of Neuroscience, Ohio State University, Columbus, OH 43210, USA

## 

Neurofilaments, one of cytoskeletal filaments determining the axonal calibers, are transported by molecular motors along microtubule tracks in a stochastic manner named ‘stop and go’[1,2,4,7], which is called the slow axonal transport. Experiments using photo-bleached live-cell imaging observed that the slow axonal transport of NFs is characterized by a rapidly intermittent, bidirectional movement [1,3]. The overall process of brief moving interspersed by short or long term pausing can be modeled by a well-tested mathematical model [5,6], where NFs could stay on-track running, on-track pausing and off-track pausing for both anterograde and retrograde directions. The shortage of photo-bleached live-cell imaging is that it under-estimates the population of NFs which stay in the long-term pausing state. Fluorescence photo-activation pulse-escape method is designed to observe the long-term decay of the total population of NFs, which can provide accurate information about the time that NFs stay off-track. We developed a systematical method based on the model [6] and analyzed the pulse-escape experimental data in Superior Cervical Ganglion (SCG) and Dorsal Root Ganglion (DRG) neurons. The fluorescence decay of the photo-activated neurofilaments can be fit by a double exponential function, where we can extract the short-time initial decaying rate and long-term decaying rate; simultaneously, those two rates can be obtained by solving the partial differential equation which describes the stochastic movement of NFs. By using those two conditions we constrain our parameter space such that we can find a unique set of transition rates that describe the kinetics of NFs. Therefore, all the other dynamic characterization such as the average velocity of NFs, average on-track running and pausing, off-track pausing time, percentage of population of NFs at each state can all be predicted.

An important advantage of our new analytical approach is that it can permit the characterization of neruofilament transport in mature axons for which single-neurofilament tracking is optically challenging due to the thickness or abundance of neurofilaments. This combined experimental and computational approach is a powerful tool for the analysis of the moving and pausing behavior of neurofilaments in axon.
